# Mechanisms and salvage treatments in patients with multiple myeloma relapsed post-BCMA CAR-T cell therapy

**DOI:** 10.3389/fimmu.2024.1433774

**Published:** 2024-10-22

**Authors:** Bingjie Fu, Rui Liu, Gongzhizi Gao, Zujie Lin, Aili He

**Affiliations:** ^1^ Department of Hematology, The Second Affiliated Hospital of Xi’an Jiaotong University, Xi’an, China; ^2^ National-Local Joint Engineering Research Center of Biodiagnostics & Biotherapy, The Second Affiliated Hospital of Xi’an Jiaotong University, Xi’an, China; ^3^ Xi’an Key Laboratory of Hematological Diseases, Xi’an, China

**Keywords:** multiple myeloma, BCMA CAR-T, relapse, salvage treatment, T-cell -engaging therapy

## Abstract

Chimeric antigen receptor T-cell (CAR-T) therapy has ushered in a new era for the treatment of multiple myeloma (MM). Numerous clinical studies, especially those involving B-cell maturation antigen (BCMA)-directed CAR-T, have shown remarkable efficacy in patients with relapsed or refractory multiple myeloma (R/R MM). However, a considerable number of patients still experience disease recurrence or progression after BCMA CAR-T treatment, which is attributed to various factors, including antigen escape, CAR-T manufacturing factors, T cell exhaustion, inhibitory effects of tumor microenvironment and impact of prior treatments. The scarcity of effective treatment options following post-CAR-T disease recurrence, coupled with the lack of well-established salvage regimens, leaves patients who do relapse facing a bleak prognosis. In recent years, some academic institutions have achieved certain results in salvage treatments of patients with relapse after BCMA CAR-T treatment through secondary infusion of BCMA CAR-T, changing to non-BCMA-directed CAR-T, double-target CAR-T, bispecific antibodies or other novel therapies. This review summarizes the mechanisms of resistance or relapse after BCMA CAR-T administration and the available data on current salvage treatments, hoping to provide ideas for optimizing clinical salvage therapies.

## 1 Introduction

Multiple myeloma (MM), characterized by presence of abnormal clonal plasma cells, is the second most common hematological malignancy (1, 2). Over the years, the development of hematopoietic stem cell transplantation, immunomodulatory drugs, proteasome inhibitors and monoclonal antibodies has significantly improved the prognosis of multiple myeloma patients (3–7). Despite these advances, MM remains incurable and disease relapse is inevitable in most patients. Recent years, chimeric antigen receptor T-cell (CAR-T) therapies, especially those targeting B-cell maturation antigen (BCMA), a cell surface glycoprotein primarily expressed by plasma cells and some mature B cells, have shown remarkable efficacy in patients with relapsed or refractory MM (R/R MM) (8–11). To date, the US Food & Drug Administration (FDA) has approved two anti-BCMA CAR-T cell products, idecabtagene vicleucel (ide-cel) and ciltacabtagene autoleucel (cilta-cel) for the treatment of R/R MM. Some patients exhibit durable responses–for instance, in the LEGEND-2 trial, 16.2% of participants experienced lasting responses and remained relapse-free, with the longest remission being as long as 6.4 years (12). However, a considerable proportion of patients eventually experience disease relapse or progression. This underscores the necessity to explore salvage therapeutic strategies following BCMA CAR-T therapy recurrence. In this review, we summarize the mechanisms of relapse after BCMA CAR-T treatment and the current data on salvage treatment options, providing recommendations for optimal treatment strategies for patients following BCMA CAR-T relapse.

## 2 Mechanisms of resistance to BCMA CAR-T cells

**Figure 1:**
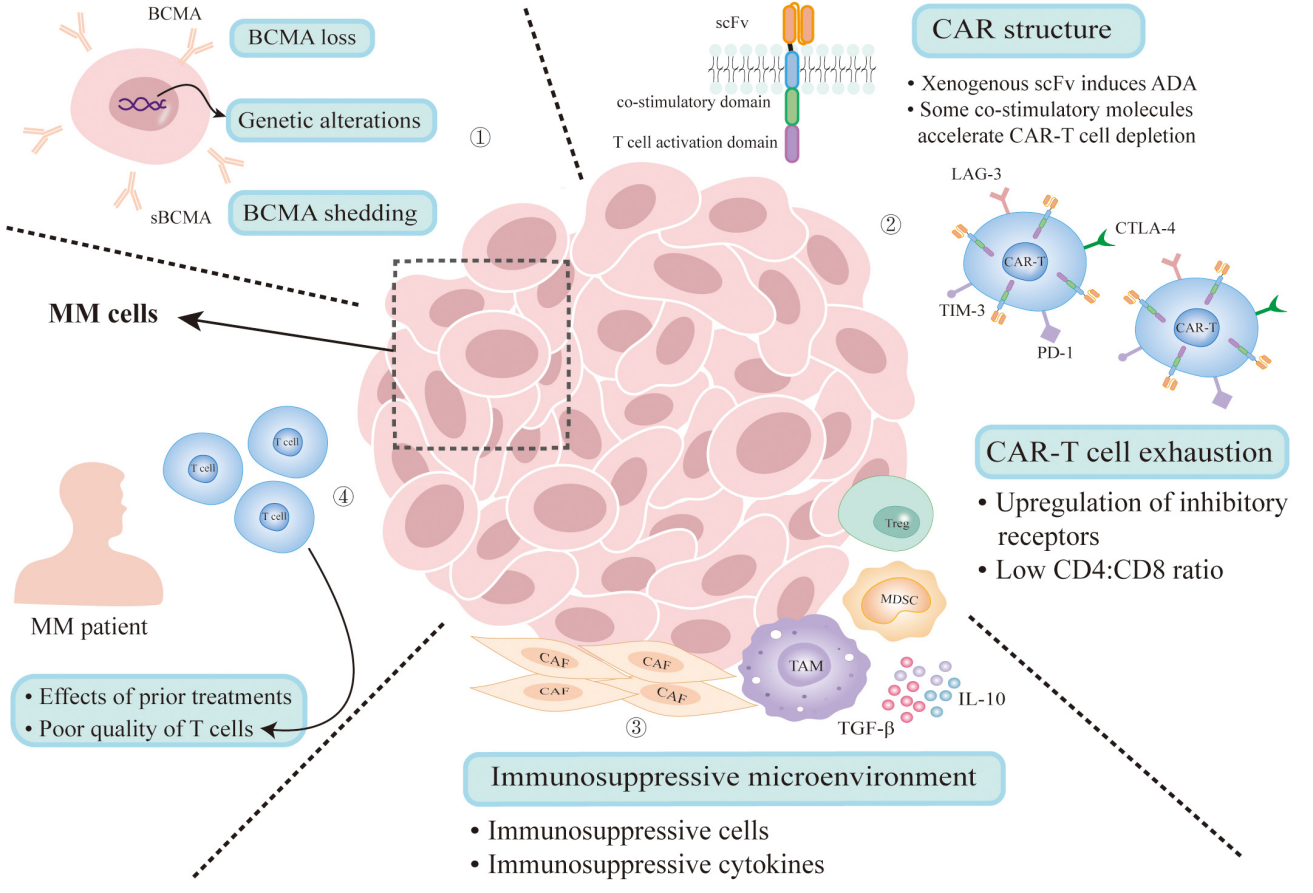
Mechanisms of resistance to BCMA CAR-T therapy. (1) Antigen escape due to genetic alterations and clonal selection under therapeutic pressure. Gamma secretase cleaves BCMA from the surface of MM cells that releases sBCMA. (2) The presence of anti-scFv antibodies results in reduced CAR-T cell count and loss of activity. Some co-stimulatory molecules can accelerate CAR-T cell depletion. CAR-T cell exhaustion results from persisting antigenic stimulation and immunosuppressive microenvironment can also lead to disease relapse. (3) Immunosuppressive microenvironment impairs CAR-T cell activation. (4) Multiple treatment regimens will impact the environment of CAR-T therapy and the quality of T cells collected from MM patients is poor. MM, multiple myeloma; BCMA, B-cell maturation antigen; sBCMA, soluble BCMA; ADA, anti-CAR antibodies; CAF, cancer-associated fibroblast; TAM, tumor associated macrophage; MDSC, myeloid-derived suppressor cell; Treg, regulatory T-cell.

Despite the encouraging achievements of anti-BCMA CAR-T therapy in R/R MM (11, 13), the issue of recurrence in MM patients is equally deserving serious attention. The resistance mechanisms are closely related to antigen escape, factors of CAR-T production, T cell exhaustion, the interaction between tumor cells and complex tumor microenvironment and the influence of previous treatment (**Figure 1**). A precise grasp of these mechanisms can help guide subsequent medical decisions and overcome the resistance.

### 2.1 Antigen escape

Compared to normal cells, BCMA is highly expressed in myeloma cells. It has been confirmed that overexpression and activation of BCMA are closely related to the progression of MM, which makes it the most attractive therapeutic target in MM (9, 14). However, MM is a highly heterogeneous malignancy with a large number of subclones in the same patient, suggesting MM may be particularly susceptible to evading single antigen-targeted therapy. Brudno et al. found that 1 in 16 patients had a loss of BCMA in myeloma cells after BCMA CAR-T cell infusion (15). Similarly, Ali et al. found that one of the eight patients who were continuously followed in their study had a BCMA-negative recurrence (16). The mechanism of these phenomena might be clonal selection under therapeutic pressure: myeloma cells with high BCMA expression are eliminated by BCMA CAR-T cells, while low BCMA expression cells are selected to survive. Over time, these antigen-negative subclones could expand and dominate the tumor population, leading to antigen loss.

In addition to clonal selection, genetic alterations could also lead to antigen loss. Samur et al. found that biallelic loss of BCMA is one of the resistance mechanisms to anti-BCMA CAR-T cell therapy by performing single-cell transcriptome profiling of bone marrow samples serially collected from a patient treated with ide-cel (17). Similarly, Lee et al. found that biallelic loss of TNFRSF17 gene encoding BCMA can result in the loss of the BCMA antigen, while extracellular domain mutations can lead to loss of functional BCMA epitopes, significantly attenuating the binding between CAR-T cells and their cognate epitopes, thereby causing disease recurrence (18). Based on the above, targeting other antigens, or constructing dual-target CAR-T, can overcome the loss of BCMA.

Furthermore, BCMA can be cleaved from the surface of MM cells by gamma secretase, releasing soluble BCMA (sBCMA), which presents in most R/R MM patients. It can not only decrease functional BCMA on the surface of MM cells, but interfere with BCMA-directed CAR-T cells (19, 20). In this case, gamma secretase inhibitors have shown promise for improving the efficacy of BCMA CAR-T therapy (21).

### 2.2 Factors of CAR-T production and T cell exhaustion

#### 2.2.1 Factors of CAR-T production

CAR-T is constructed by modifying T cells isolated from patients, thus the condition of T cells has an important impact on the durability and efficacy of CAR-T. One study found that apheresis samples from patients with earlier stages of MM exhibited a higher percentage of memory T cells and CD4/CD8 ratio than those from R/R MM patients, suggesting that T cells from patients with early treatment stage are more suitable for CAR-T manufacturing and CAR-T cells may be more effective if they are manufactured from patients before onset of recurrent/refractory (22).

The structural design of CAR molecules determines the property of CAR-T cells. The co-stimulatory domain, an important component of CAR, presenting in the second-generation and later CAR-T products, significantly affects the function of CAR-T cells, such as depletion rate, anti-tumor activity and persistence (23, 24). CD28, 4-1BB, ICOS (CD278), OX40 (CD134) are the most commonly used co-stimulatory molecules. CD28 triggers powerful T cell activation but accelerates CAR-T cell depletion. In the contrary, 4-1BB can improve CAR-T cell depletion and promote the expansion of stem cell memory T cells. Besides, the combination of ICOS and 4-1BB can significantly improve the persistence of CAR-T cells. OX40 shows the ability to promote T cell proliferation and memory formation (23, 25–28). In the view of the importance of co-stimulatory factors and their existing shortcomings, new co-stimulatory molecules or combination structures are still being explored to improve CAR-T function.

In addition, the emergence of anti-CAR antibodies may be another important factor leading to CAR-T resistance. Most BCMA-targeting CAR-T cells have derived their scFv from mouse or other non-human species. Xenogenous scFv can induce the appearance of anti-CAR antibodies (ADA), resulting in reduced CAR-T cell count and loss of activity (29–31). By analyzing 17 R/R MM patients treated with LCAR-B38M, Xu et al. found that 6 out of 7 patients with relapse or disease progression showed high levels of ADA and their residual CAR-T cells in peripheral blood were deeply reduced. This suggests that ADA may pose a high risk of relapse after treatment (32). A study of ADA testing of 55 R/R MM patients participating in LCAR-B38M treatment found that 34 (62%) were ADA positive, and the frequency of ADA in cyclophosphamide plus fludarabine group was lower than that in cyclophosphamide group, suggesting the choice of lymphodepleting regimen has an important influence on ADA generation. However, the presence of ADA had no effect on overall survival (OS), progression-free survival (PFS), or duration of response (DOR) (33).The impact of ADA presence on BCMA CAR-T efficacy remains controversial. Additional data is necessary to confirm these observations, and strategies to develop whole humanized scFv or antigen-recognition domains composed of fully humanized pure heavy chain antibody variable domains may overcome the potential adverse impact generated by ADA (29, 34–36), advancing the efficacy of CAR-T cell therapy.

#### 2.2.2 T cell exhaustion

Persisting antigenic stimulation and immunosuppressive tumor microenvironment can cause T cell exhaustion, defined as the inhibition of T cell proliferation and effector function (37–39). After BCMA CAR-T cell infusion, the senescence phenotype in CD8-positive T cells increased more than that in CD4-positive T cells, and an increase in the CD4:CD8 ratio in the infusion products was associated with better amplification and clinical outcomes (15, 40). In addition, Ledergor et al. found that patients exhibiting durable responses to CAR-T cell therapy possessed a significantly higher proportion of CD8+ T-effector memory cells. Conversely, in patients with transient responses to treatment, there was an increase in cytotoxic CD4+ CAR-T cells, which expanded early *in vivo* after infusion but expressed exhaustion markers while maintaining polyclonality. Furthermore, they discovered that myeloid cells in the myeloma niche may contribute to CD4+ CAR-T cell dysfunction via transforming growth factor beta (TGF-β) (41).

Increased expression of multiple inhibitory receptors is another feature of T cell depletion, including programmed cell death protein 1 (PD-1), cytotoxic T lymphocyte antigen 4 (CTLA-4), T-cell immunoglobulin domain and mucin domain-3 (TIM-3), lymphocyte activation gene 3 (LAG-3), etc. (42, 43). It has been found in a variety of cancers that the expression of these inhibitory receptors is up-regulated in CAR-T cells of patients who have failed CAR-T therapy (42, 43). Previous studies have demonstrated that the deletion of the inhibitory co-receptor CTLA-4 and the blocking of PD-1 can enhance CAR-T function (44, 45). In MM, researchers added an anti-PD-1 short hairpin RNA to BCMA CAR-T cell, and it has showed reduced T-cell exhaustion and increased percentage of memory T cells *in vitro* (46), improving the efficacy of CAR-T cell therapy.

### 2.3 Immunosuppressive tumor microenvironment

MM microenvironment contains many tumor-supporting and immunosuppressive components, such as myeloid derived suppressor cells (MDSCs), regulatory T-cells (Tregs), tumor-associated macrophages and a variety of immunosuppressive cytokines (such as IL-10, TGF-β, etc.) (47, 48). A study has revealed that MM patients treated with BCMA CAR-T cells developing macrophage-activated syndrome-like manifestations, leading to lower 1-year survival rates and progression-free survival times (49). Li et al. conducted a single-cell sequencing on a patient who relapsed for the first time after BCMA CAR-T treatment 22 months later. They found that immune cells including T cells, natural killer (NK) cells, dendritic cells (DC), neutrophils, and monocytes/macrophages showed tumor-promoting phenotypes when disease relapsed (50), revealing tumor microenvironment is one of the underlying mechanisms of R/R MM recurrence after BCMA CAR-T cell therapy. Sakemura et al. revealed that a high presence of cancer-associated fibroblasts (CAFs) can suppress the anti-tumor activity of CAR-T cells. These CAFs concurrently produced elevated levels of inhibitory cytokines, such as TGF-β, and upregulated the surface expression of inhibitory ligands such as programmed cell death-ligand 1 (PD-L1). Additionally, they observed a significant increase expression of PD-1 on CAR-T cells, which contributes to their functional impairment. Dual-specific CAR-T cells targeted against both MM cells and CAFs were capable of overcoming the suppressive effects imposed by CAFs, thereby enhancing the anti-tumor efficacy of the CAR-T cell therapy (51).

### 2.4 Influence of previous treatment regimens

Most R/R MM patients have received multiple treatment regimens prior to BCMA CAR-T therapy (52, 53), and these regimens may impact the efficacy and duration of BCMA CAR-T cell. Studies have shown that the use of alkylating agents such as cyclophosphamide and bendamustine prior to leukapheresis could lead to the depletion of T cells and was associated with poor CAR-T manufacturing, particularly detrimental for autologous CAR-T therapies (54–56). Additionally, Ramos et al. found that patients receiving a fludarabine-based lymphodepletion had more durable responses to CAR-T therapy compared to those receiving bendamustine alone (57), which may explain why bendamustine is used less frequently as a lymphodepleting chemotherapy compared to fludarabine/cyclophosphamide regimen (58). Another study revealed that patients treated with daratumumab as bridging therapy had better response rates after CAR-T cell transfusions compared to patients who did not receive daratumumab. At the same time, daratumumab has immunomodulatory effects on the bone marrow tumor microenvironment, thereby reducing environmental resistance (8, 59). Moreover, there are now two BCMA CAR-T products approved by the FDA for the treatment of early-stage multiple myeloma. Therefore, it is advisable to select appropriate preconditioning regimens and effective less toxic bridging therapies prior to CAR-T treatment based on the patients’ situation, with the goal of optimizing the outcomes of CAR-T therapy.

## 3 Salvage treatment strategies for MM patients after BCMA CAR-T relapse

Relapse after anti-BCMA CAR-T cell therapy has been common, with more than 50% of patients having relapsed after CAR-T cells infusion (12, 60, 61). Our recently updated 5-year follow-up results of LEGEND-2 trial demonstrated that 71.6% of patients appeared disease progression (PD) after partial response (PR) or better (12). Most patients before treated with BCMA CAR-T have undergone rigorous preconditioning, thus subsequent therapy options remain limited. There is currently a lack of recommended salvage therapy for patients who relapse after CAR-T cell therapy. Here, we introduce and summarize the existing follow-up treatments after recurrence of CAR-T treatment, with selected trials highlighted in **Table 1**, so as to provide reference for improving the prognosis of patients who progress after CAR-T treatment.

**Table 1:** Treatment efficacy in multiple myeloma patients with disease relapse after BCMA CAR-T cell therapy.

Agents	No. Pts	Target	ORR	Clinical Remission	Reference
Idecabtagene vicleucel	28	BCMA CAR-T	21%	VGPR: 4%; PR: 18%; SD: 18%;	Munshi et al. (62)
Idecabtagene vicleucel	5	BCMA CAR-T	100%	≥CR: 60%; VGPR: 20%; PR: 20%	Ferreri et al. (63)
OriCAR-017	5	GPRC5D CAR-T	100%	sCR: 60%; VGPR: 40%	Zhang et al. (64)
	9	GPRC5D CAR-T	100%	CR: 44.4% VGPR: 22.2% PR: 22.2%	Xia et al. (65)
	3	GPRC5D CAR-T	67.7%	CR: 33.3%; PR: 33.3%; NR: 33.3%	Li et al. (66)
CS1-BCMA CAR-T	2	CS1-BCMA CAR-T	50%	VGPR:50%; NR:50%	Li et al. (67)
BC19 CAR-T	5^✧^	CD19/BCMA CAR-T	60%^✧^	^✧^sCR: 40% PR: 20% SD: 40%	Shi et al. (68)
Teclistamab	21	BCMA BsAb	33.3%	Near CR and CR: 15.8%; VGPR:5.2%; PR: 10.5%	Riedhammer et al. (69)
Talquetamab	16	GPRC5D BsAb	50%		Chari et al. (70)
Cevostamab	9	FCRH5 BsAb	44.4%		Trudel et al. (71)
Modakafusp Alfa	8	Anti-CD38 immunocytokine	27%*	≥VGPR: 13%*	Dan et al. (72)
Nivolumab	11	PD-1 monoclonal antibody	18%	VGPR: 18%	Banerjee et al. (73)
Selinexor	7	XPO1 inhibitor	86%	sCR: 14.3%; VGPR: 42.9%; PR: 2.6%; MR: 14.3%	Chari et al. (74)

^✧^Includes patients who relapsed after CD19 or GPRC5D-targeted CAR-T therapy.

*Includes patients who received BCMA antibody-drug conjugate (ADC).

ORR, overall response rate; CR, complete response; sCR, stringent CR; PR, partial response; VGPR, very good PR; SD, stable disease; MR, minimal response.

### 3.1 Re-infusion of BCMA CAR-T products

Disease progression after BCMA CAR-T treatment is partly due to the short duration of CAR-T cells *in vivo* result from CAR-T product factors. Therefore, secondary infusion of the same BCMA CAR-T product or full human-derived BCMA CAR-T can be performed to improve the persistence of CAR-T *in vivo* and thus improve the survival prognosis of patients.

Munshi et al. conducted a single-group phase II study of ide-cel which a total of 128 patients received ide-cel infusions. After PD, 28 patients received ide-cel reinfusion and 6 patients (21%) had a second response, with durations of response ranging from 1.9 to 6.8 months. It is noting that all the patients who had a response were retreated with a higher dose than their initial dose (62). This suggests that the same CAR-T product has limited efficacy as a salvage treatment for patients who relapse after BCMA CAR-T therapy.

In a retrospective study of R/R MM patients treated with commercial ide-cel, five of the patients had previously undergone BCMA CAR-T therapy. Two of them had received ide-cel as their prior CAR construct in early phase trials, two had received an allogeneic CAR, and one had received an autologous CAR with a non-viral transduction. This subgroup had the best response rate to ide-cel (overall response rate [ORR]: 100%; ≥complete response [CR]: 60%) (63). A clinical trial of BCMA CAR-T with a fully human scFv (CT103A) enrolled 18 consecutive adult subjects with BCMA-positive R/R MM, including 4 with prior murine BCMA CAR exposures. The ORR was 100%, and 72.2% of patients achieved CR or stringent CR (sCR). For the 4 murine BCMA CAR–exposed patients, 3 achieved sCR, and 1 achieved a very good partial response (VGPR) (36). Similarly, a study reported that two patients with PD after infusion of murine CAR-T, including 1 patient with extramedullary disease (EMD) at PD, achieved VGPR and PR after rescue therapy with humanized BCMA CAR-T cells (75). In another clinical trial of fully human anti-BCMA CAR-T (HRC0202), 7 R/R MM patients previously exposed to anti-BCMA CAR-T therapy were evaluated. The ORR was 71.4% (5/7), and 3 patients achieved sCR/CR. The median PFS (mPFS) was 269 days (76). These studies indicate that fully human anti-BCMA CAR-T is a promising treatment for R/R MM patients who relapsed or refractory from prior non-human-derived anti-BCMA CAR-T infusion.

### 3.2 Switching to non-BCMA-directed CAR-T cell therapy

Patients with antigen loss or low, variable BCMA expression may benefit from new target CAR-T therapies, particularly CAR-T directed at G protein–coupled receptor class C group 5 member D (GPRC5D), which presents as an appealing alternative. Previous studies have shown that GPRC5D was highly expressed on myeloma cells. Except bone marrow, GPRC5D expression is observed in keratin synthesis, lung tissue, and the inferior olivary nucleus (77, 78). In addition, GPRC5D is a seven-pass transmembrane receptor protein, so it is unlikely to be shed in the serum, greatly reducing the risk of disease recurrence due to antigen shedding (79, 80). Besides, expression of GPRC5D is independent of BCMA (79), making GPRC5D CAR-T capable of killing MM cells in spite of BCMA expression. In a phase 1 dose-escalation study, 17 R/R MM patients received anti-GPRC5D CAR-T cell infusion. Ten had previously received BCMA-targeted therapy, of which eight had received BCMA CAR-T therapy. Responses were observed in 7 of 10 patients who had received previous BCMA therapies (81). In a single center, phase 1 trial, 10 R/R MM patients were treated with GPRC5D CAR-T cells (OriCAR-017), and five of them had been treated with BCMA CAR-T. 100% of ORR was observed, with 60% (6/10) of them achieving sCR (64). Recently, at 2023 American Society of Hematology (ASH) annual meeting, a clinical trial of BMS-986393, a GPRC5D targeted CAR-T product, 75% (21/28) of ORR was observed in patients treated with prior BCMA therapies, including BCMA CAR-T cells (NCT04674813) (82). From the above results, we can see that GPRC5D is an active target in multiple myeloma and has also responded well in patients who had failed in prior anti-BCMA therapy.

### 3.3 Rescuing with dual-target CAR-T cells

Compared to single-target CAR-T cells, double-target CAR-T cells increase the coverage of antigen, decrease the possibility of myeloma relapse by BCMA-negative clones, thus have better anti-myeloma effect (83–85).

CS1, also known as CD319 and SLAMF7, is expressed at high levels on multiple myeloma cells (86, 87). Li et al. found that the MM cells of relapsed patients after anti-BCMA CAR-T cell therapy with BCMA loss still maintained CS1 expression. Therefore, they constructed a bispecific CS1-BCMA CAR-T cell to augment BCMA targeting with CS1. Fourteen patients who had not received BCMA CAR-T and two who had relapsed after BCMA CAR-T cell therapy, received CS1-BCMA CAR-T cell transfusions. The ORR was 81%. Of the two patients who received prior BCMA CAR-T therapy, one obtained VGPR but the other one with solitary EMD did not respond (67).

Researchers found that although CD19 is only expressed on a subset of MM cells, these cells are typically less differentiated and possess clonogenic capacity, considered to be poorly differentiated MM cells or myeloma-like stem cells (88, 89). Even with low CD19 expression, these cells can still be eliminated by CD19 CAR-T cells (89, 90). Therefore, therapies targeting this subpopulation may have synergistic effects with other treatments, helping to eliminate less differentiated myeloma cells. Based on the above, Shi et al. designed bispecific BC19 CAR-T cells targeting BCMA/CD19 and evaluate antimyeloma activity in 50 patients. Five patients in this study had received prior BCMA, CD19, or GPRC5D-targeted CAR-T cell treatment. Two of them achieved sCR, one achieved PR, and the other two had stable disease (SD) (68). These studies suggest that dual-target CAR-T has promising clinical activity in R/R MM patients even after BCMA CAR-T cell treatment.

### 3.4 Using bispecific antibody or combining bispecific antibody with monoclonal antibody

Bispecific antibodies (BsAb) are designed to bind to targets on tumor cells and cytotoxic immune effector cells (T cells/NK cells) to create immune synapses that cause T/NK cells to activate and direct them to attack and destroy tumor cells (91, 92).

Teclistamab is a BCMA×CD3 directed bispecific antibody. In the C cohort of MajesTEC-1 trial, 11 of the 25 evaluable patients had previously received BCMA CAR-T. They achieved an ORR of 45% after treated with teclistamab, and their responses deepened over time (93). A retrospective study analyzing 123 patients treated with teclistamab found that among the 21 patients previously treated with BCMA CAR-T, the ORR was only 33.3%, with a significantly shorter median PFS of 1.8 months. Furthermore, the duration of response to teclistamab in patients achieving an overall response did not differ from those who had not received prior BCMA CAR-T therapy (69). These suggest a lower response rate to teclistamab after BCMA-directed CAR-T cell therapy. However, another retrospective analysis of teclistamab included 106 R/R MM patients, of whom 56 had previously received BCMA-directed therapy, including 42 who had previously undergone BCMA CAR-T treatment. These patients achieved response rates comparable to those seen in the overall patient population (ORR: 59% vs 66%) (94), while the long-term efficiency outcome was not known.

Talquetamab, a bispecific antibody that against GPRC5D and CD3, recruits and activates T cells to kill GPRC5D-expressing myeloma cells (70, 95). In a study presented at 2023 ASH meeting, 50 of 70 patients with CAR-T exposure (48/50 anti-BCMA CAR-T) were treated with talquetamab. Among them whose efficacy could be assessed, their ORR was 72.9%, similar to the overall population (96). This supports that talquetamab can serve as a multifunctional treatment option to provide effective responses in BCMA CAR-T exposed R/R MM patients.

Fc receptor-homolog 5 (FcRH5), also known as CD307, is a membrane protein. It is only expressed on B cell lineage and at a higher level on myeloma cells than normal B cells. Cevostamab is a FcRH5×CD3 BsAb that facilitates T cell-directed killing of myeloma cells. Of the patients participating in the cevostamab monotherapy study, 9 patients who had received BCMA were efficacy evaluable and they achieved an ORR at 44.4% (71).

In addition to BsAb, the combination of BsAb and monoclonal antibody (mAb) is also another choice of rescue measures. Daratumumab, a CD38 mAb, has shown a good safety and efficacy profile when used in combination with other anti-myeloma drugs (97, 98). In the phase 2 TRIMM-2 trial (NCT04108195), 65 R/R MM patients were enrolled to receive talquetamab plus daratumumab. Prior treatments included anti-CD38 (88%), anti-BCMA (54%), BsAb (25%), and anti-BCMA CAR-T (17%) therapy. In patients exposed to prior anti-BCMA therapy, ORR was 74%. At 12 months, 86% of the responders (89% of patients ≥CR) were still responding (99). These results suggest that combining BsAb with anti-CD38 mAb can yield robust responses in patients who have relapsed after BCMA CAR-T.

### 3.5 Other novel therapies

Modakafusp alfa is an immunocytokine fusion protein combining attenuated interferon alpha (IFNɑ) to be molecules attached to an anti-CD38 IgG4 mAb, delivering IFNɑ to CD38-expressing cells such as myeloma cells. Among 30 patients treated with modakafusp 1.5 mg/kg Q4W, 16 patients (50%) had previously received anti-BCMA therapy, of which 8 (27%) had received CAR-T therapy. The ORR was 43% and the median PFS was 5.7 months. In patients with prior anti-BCMA therapy (half received BCMA CAR-T cell therapy), the ORR was 27%, of which 13%≥VGPR (72).

Checkpoint inhibitors can theoretically improve CAR-T cell activity. To characterize the efficacy of nivolumab (a monoclonal antibody targeting PD-1 on T-cells) in MM after CAR-T failure, Banerjee et al. enrolled 11 patients. After nivolumab monotherapy, their ORR was 18% (2/11) (73). However, this is only a prospective study with a small number of patients included in the study. More trials are needed to verify the efficacy of PD-1 monoclonal antibody in BCMA CAR-T relapsed populations.

In addition to salvage with T-cell–engaging therapies after CAR-T, other saving methods or multiple follow-up treatments can be used based on patient characteristics. Selinexor is an oral small molecule nuclear export protein export protein 1 (XPO1) inhibitor that promotes apoptosis in malignant tumor cells such as myeloma cells (100, 101). The activity of selinexor was preserved regardless of prior therapy. Therefore, Chari et al. identified seven patients who had progressed after BCMA CAR-T to receive a selinexor combination immediately after CAR-T progression and the responses included 1 sCR, 3 VGPR, 2 PR and 1 minimal response (74). While the findings reported here are derived from a small cohort of patients, the trial demonstrated the anti-myeloma activity of selinexor irrespective of prior treatment history and without evidence of cross-resistance. Adenosine has a highly immunosuppressive effect, and CD73 can catalyze adenosine monophosphate (AMP)-producing adenosine. ORIC-533 is a small molecule inhibitor of CD73 that inhibits CD73 to block adenosine production to restore and enhance immune function. A recent study demonstrated a preliminary evidence of immune activation after ORIC-533 in heavily pretreated R/R MM patients. Seventeen patients were treated with four dose levels of ORIC-533, 59% of whom had previously received BCMA/CD3 bispecific therapy or anti-BCMA CAR-T therapy. After one treatment cycle, an increase in the abundance of circulating NK and CD8+ T cells was observed (102).

Radiotherapy (RT) is a local control method. A study involved 13 patients with locally progressive bone or soft tissue plasmacytomas. Five of them only received bridging RT before CAR-T, four received salvage RT after CAR-T failure, and another four received both bridging and salvage RT. The median overall survival of the cohort was 16.2 months, the local control rate was 100%, and no radiation-related toxicity was observed (103). Iopofosine I-131 is a phospholipid radioactive conjugate that causes apoptosis by causing double-stranded DNA breaks. The six patients receiving a total dose >60 mCi had an ORR of 50% and the clinical benefit (stable or better disease) of 100% (104). In addition, two case reports introduced the clinical effects of myeloablative monophasic hematopoietic stem cell transplantation and triple MAPK inhibition strategies for saving BCMA CAR-T recurrence, indicating their feasibility (105, 106).

## 4 Comparison of the efficacy of different salvage therapies in one cohort

Chen et al. first reported the outcomes of 20 patients who received subsequent anti-myeloma therapies (sAMT) following progression on HDS269B, a BCMA CAR-T. Subsequent treatments included anti-CD38 mAb-based therapy, pomalidomide-based therapy, daratumumab-based therapy, and further rounds of CAR-T. Nine patients (45%) went on to receive more than three sAMTs. Apart from three patients who could not be evaluated, the ORR to the first sAMT was 35%, and the clinical benefit rate was 65% (60).

Van et al. analyzed the salvage treatments and outcomes of 79 R/R MM patients from two academic institutions who had progression of disease after treatment with BCMA CAR-T. The ORR for first-line salvage therapy was 43.4% and the median PFS was 3.5 months. Median overall survival was 17.9 months after multiline salvage therapy and researchers did not find ORR to decrease significantly up to beyond the fifth line of salvage therapy. The first-line treatment options include CAR-T Trials $\left(n=2\right)$ , non-BCMA-directed bispecific trial $\left(n=9\right)$ , venetoclax-based therapy $\left(n=3\right)$ , chemotherapy $\left(n=20\right)$ , BCMA-directed bispecific trial $\left(n=2\right)$ , selinexor-based therapy $\left(n=5\right)$ , autologous hematopoietic stem cell transplantation (Auto-HSCT) $\left(n=3\right)$ , doublet/triplet/quadruplet combination of approved agents $\left(n=23\right)$ , other in combinations (including MAPKi, checkpoint inhibitor or other trial) $\left(n=11\right)$ and BCMA antibody-drug conjugate (ADC) $\left(n=1\right)$ , the ORRs of these schemes were 100%, 75%, 66.7%, 57.9%, 50%, 40%, 33.3%, 31.8%, 9.1% and 0%, respectively (61). This study indicates that though the response rate and/or duration of each salvage treatment option after BCMA CAR-T recurrence is modest, there are a wide range of management strategies can be used as salvage anti-myeloma treatment options.

Reyes et al. analyzed 78 R/R MM patients who received BCMA CAR-T therapy. Of 42 patients with post-CAR-T PD, 37 (88%) received ≥1 follow-up salvage treatment with a median of 3 (range: 1-8) lines. ORR in patients who subsequently received BCMA CAR-T, BCMA-targeted BsAb, anti-CD38 antibodies, alkylators, proteosome inhibitors (PIs), or immunomodulatory drugs (IMiDs) as salvage therapy was 75%, 60%, 52.6%, 46.3%, 40.6% and 37.5%, respectively. In addition, they found that patients who had previously failed to respond to a specific drug class prior to CAR-T were able to show a renewed response after CAR-T relapsed (107). In the updated follow-up from last year, 58 patients received salvage therapy, and first-line salvage strategies included alkylating agent therapy, CD38-based combination therapy, and BCMA-directed alternative therapy. The ORR for first-line salvage therapy was 41%. The ORR of the other BCMA-directed CAR-T was 89% and the ORR of the BCMA-directed BsAb was 60% of all salvage treatment lines. In CD38-oriented combinations, ORR was 80% when combined with BsAb and the ORR was 44% in combination with IMiD and/or PI. The alkylator-based therapies were also effective with an ORR of 50% (108). Indeed, in their cohort, T-cell engaging rescue therapies appeared to be more effective. A recent retrospective study explored the prognosis and optimal treatment sequence for patients who relapsed after BCMA CAR-T or double antibody therapy. Among the 38 patients studied, 17 were in the CAR-T group and 21 in the BiAbs group, all of whom received salvage therapy after relapse. To identify the optimal treatment sequence, these patients were divided into four groups: CAR-T followed by BiAbs, CAR-T followed by other schemes, BiAbs followed by other BiAbs and BiAbs followed by other schemes. Results showed that nearly 90% of patients in the CAR-T followed by BiAbs group achieved at least a partial response (≥PR), with 38% achieving at least a complete response (≥CR). In contrast, the other three groups had lower ORR ranging from 30% to 50% and fewer instances of deep remission. Besides, the treatment sequence of CAR-T followed by BiAbs also produced a longer mPFS than CAR-T followed with other schemes, suggesting BiAbs could be an optimal therapeutic option after BCMA CAR-T (109).

In our recent retrospective analysis of R/R MM patients treated with LCAR-B38M enrolled in the LEGEND-2 trial showed that 34 (76%) patients had evidence of PD and 32 patients received salvage therapy, including PI-based combination therapy $\left(n=15\right)$ , second BCMA CAR-T $\left(n=10\right)$ , and combination chemotherapy $\left(n=7\right)$ . The ORR for this cohort was 50.0% with a median PFS was 16.3 months. In addition, the analysis found that despite 67% of the patients who received PI-based combination salvage therapy having been previously exposed to PI, this salvage therapy had a significantly higher ORR (80%) compared to second CAR-T infusion (30%) and chemotherapy (0%) (110).

## 5 Conclusion and prospective

In recent years, anti-BCMA CAR-T cell therapy has achieved impressive results in R/R MM patients with generally manageable side effects. However, certain challenges persist, such as the occurrence of recurrences even after anti-BCMA CAR-T cell therapy. Indeed, standards or recommended guidelines for post-CAR-T salvage therapy have not yet been established, highlighting a significant area of unmet need in multiple myeloma treatment. In this review, after summarizing the data of various salvage therapies, it appears that the efficacy of BCMA CAR-T therapy as a salvage therapy after prior BCMA CAR-T relapse seems to be limited, and other T-cell–engaging therapies, such as CAR-T targeting other targets, dual-target CAR-T, bispecific antibodies, etc., demonstrate notable clinical activity, with high response rate and long-lasting response. Currently, the data on dual-target CAR-T therapy for salvage treatment after BCMA CAR-T relapse primarily focus on combinations of BCMA with CS1 or CD19 (67, 68). Ongoing research is exploring the effectiveness of combining BCMA with other targets such as TACI, GPRC5D, CD38, CD24, and more. Clinical trials involving these combinations have shown promising anti-myeloma activity (111–115), indicating their potential to rescue patients experiencing recurrence in the future.

In addition, there are other molecules expressed on the MM cell surface that can be used as CAR-T targets, such as GPRC5D. Most CD138+ cells express both BCMA and GPRC5D, but the expression of the two is independent of each other. And the antigen escape risk of GPRC5D is very low, which makes it a hot research direction in recent years (77). CAR-T targeting CD38, Natural Killer Group 2D (NKG2D) ligand, FcRH5, and kappa light chains are also under continuous development and are expected to be a promising outcome for rescue therapy (116–120). Besides, patients are disassociated from previous therapeutic agents such as PIs and IMiDs during BCMA CAR-T therapy. MM resistant cells could be targeted and eliminated by CAR-T cells, potentially allowing patients to resume their original regimen following CAR-T relapse. Moreover, CAR-T therapy may have a positive effect on bone marrow microenvironment (110). New anti-myeloma drugs such as selinexor and carfilzomib also provide a new salvage option.

On the other hand, consolidate/maintain the efficacy of CAR-T after CAR-T cell infusion to achieve a longer duration of remission may provide potential clinical benefits for high-risk MM patients. One study reported a patient whose disease continued to progress after multiple chemotherapy regimens, mouse anti-BCMA CAR-T, and human anti-BCMA CAR-T. But he achieved VGPR lasting for more than 8 months after human-derived anti-BCMA CAR-T combined with lenalidomide regimen, indicating that lenalidomide as maintenance therapy increases the expansion and efficacy of anti-BCMA CAR-T (121). Besides, Zhao et al. found that pomalidomide maintained T cell viability, promoted cell proliferation and significantly enhanced TNF-ɑ and IFN-γ secretion *in vitro* studies. Three R/R MM patients with EMD achieved 100% ORR after receiving the combination therapy (122). Similarly, a prospective study presented at the ASH meeting in 2023 reported that long-term oral administration of pomalidomide (4 mg/day) after BCMA CAR-T cell infusion can enhance the efficacy of BCMA CAR-T cell therapy, prolong PFS, and reduce the recurrence rate in patients with R/R MM (123). The above findings indicate that combination with immunomodulator can effectively enhance the efficacy of CAR-T. Certainly, the application of the combination therapy still requires strict clinical trial verification.

In conclusion, conducting long-term follow-up and larger studies is imperative to elucidate the role of these new therapies following BCMA CAR-T therapy. This will help determine the optimal sequence and combination regimen for treating relapsed/refractory multiple myeloma patients after CAR-T therapy relapse (124). Furthermore, with BCMA CAR-T now approved for earlier lines of treatment, the goal of post-CAR-T therapy should extend beyond merely rescuing patients from imminent death. Instead, the focus should be on strategically planning treatments to further prolong patient survival. Continued exploration of more effective options for managing relapse is equally essential. Ultimately, CAR-T therapy will become an even more powerful weapon against multiple myeloma.
